# Association of work-family conflict with turnover intention among hospital ophthalmologists in Japan: a cross-sectional study

**DOI:** 10.1007/s10384-025-01275-3

**Published:** 2025-09-08

**Authors:** Yukiko Tsubota, Atsushi Miyawaki, Masashi Izumiya, Yuho Shimizu, Hideo Yasunaga, Makoto Aihara, Masato Eto

**Affiliations:** 1 Department of Ophthalmology, Graduate School of Medicine, The University of Tokyo, Bunkyō, Tokyo Japan; 2 Department of Medical Education Studies, International Research Center for Medical Education, Graduate School of Medicine, The University of Tokyo, Bunkyō, Tokyo Japan; 3 Department of Health Services Research, Graduate School of Medicine, The University of Tokyo, Bunkyō, Tokyo Japan; 4 Public Health Research Group, Institute of Medicine, University of Tsukuba, Tsukuba, Ibaraki Japan; 5 Faculty of Social Innovation, Department of Psychological and Sociological Studies, Seijo University, Setagaya, Tokyo Japan; 6 Department of Clinical Epidemiology and Health Economics, School of Public Health, The University of Tokyo, Bunkyō, Tokyo Japan

**Keywords:** Turnover intention, Work–family conflict, Gender bias, Hospital, Ophthalmology

## Abstract

**Purpose:**

To examine the associations between work–family conflict, implicit gender bias, and turnover intention among hospital ophthalmologists.

**Study design:**

Cross-sectional study.

**Methods:**

We conducted a web-based questionnaire survey between January and February 2024. The participants were full-time ophthalmologists working in 37 hospitals in the Kanto region of Japan. We assessed the associations between work–family conflict, implicit gender bias, and turnover intention using multivariable regression analysis, with adjustments for job fit and position.

**Results:**

We analyzed data from 74 ophthalmologists (mean age: 41.5 years, standard deviation: 10.8; 51.4% women). The median intention to leave score was 2.50 (interquartile range: 2.00–3.00). Work interference with family was significantly associated with turnover intention (adjusted difference: 0.30; 95% confidence interval: 0.075–0.53; *p*=0.010). However, family interference with work and implicit gender bias were not significantly associated with turnover intention.

**Conclusions:**

This is the first study to examine the associations between work–family conflict, implicit gender bias, and turnover intention among Japanese hospital ophthalmologists. Work interference with family was associated with increased turnover intention but not with family interference with work or implicit gender bias.

**Supplementary Information:**

The online version contains supplementary material available at 10.1007/s10384-025-01275-3.

## Introduction

A well-functioning society requires a sufficient number of qualified physicians to maintain public health. However, the ongoing shortage of hospital physicians continues to be a pressing issue [1]. In Japan, hospital physicians consistently face long working hours and manage substantial workloads. In addition to providing patient care, they are responsible for education, research, and administrative tasks [2]. Among the 19 core specialties recognized by the Japanese Medical Specialty Board, ophthalmology has the second-lowest proportion of hospital physicians (37.5%) [3].

Today, the prevalence of dual-earner families and increasing working hours have made balancing work and family responsibilities a critical issue for both men and women [4]. A systematic review identified several factors associated with hospital physician turnover, including low job satisfaction, burnout, work-related stress, gender, excessive working hours, younger age, and work–family conflict (WFC) [5]. WFC is defined as an inter-role conflict that arises when individuals struggle to manage the pressures of both work and family life [6].

It has been asserted that ophthalmologists experience particularly high workplace pressure, with burnout rates higher than other medical specialties [7]. Work-related stress among ophthalmologists is often linked to excessive workloads, as demand exceeds capacity, leading to poor work–life balance [8]. Over the past two decades, WFC has received increasing attention in work sciences [9], and studies have found that WFC is associated with turnover intention [6, 10, 11]. The theoretical foundation for this relationship is the scarcity hypothesis, which posits that individuals have limited energy. When individuals take on multiple roles, their energy becomes depleted, leading to stress and inter-role conflicts [6, 12].

Furthermore, research reports a positive correlation between WFC and implicit gender bias [13]. In a survey on gender bias conducted by the Japanese government, 48.7% of men and 44.9% of women agreed with the statement, “Men should work and support the household financially” [14]. In Japan, women frequently leave their jobs to care for their children before they can establish sustainable careers [15]. However, research on hospital physician turnover rarely focuses on non-work-related factors, such as household chores, childcare, and caregiving [5].

Although previous studies have explored the relationship between WFC and turnover intention [5, 9] and the link between WFC and implicit gender bias [13], few have examined the relationship between turnover intention and implicit gender bias among ophthalmologists. This is particularly relevant in the Japanese context, where cultural gender biases remain significant, as reflected in the country’s comparatively low ranking on the global Gender Gap Index [16]. Therefore, this study examines the associations between WFC, implicit gender bias, and turnover intention among ophthalmologists in Japanese hospitals.

## Methods

### Study design and participants

This cross-sectional study was conducted between January and February 2024. The participants were full-time ophthalmologists affiliated with the Department of Ophthalmology at the University of Tokyo, working at seven university-affiliated hospitals and 30 general hospitals in the Kanto region, Japan. Eligible participants included board-certified ophthalmologists and ophthalmology residents in training.

After excluding physicians whose email addresses were not registered with the Department of Ophthalmology and those to whom the survey could not be sent, we distributed a web-based questionnaire to 128 potential participants on January 10, 2024. The survey assessed turnover intention, WFC, implicit gender bias, sociodemographic characteristics, and personal details. Informed consent was obtained at the beginning of the survey, and participants who did not provide consent were unable to proceed further. Those who completed all the questions received a 3,000 Japanese yen gift card, with acceptance being optional.

We sequentially excluded participants who: (i) did not provide consent, (ii) did not complete the questionnaire, (iii) reported working fewer than 32 hours per week at their primary hospital [17], and (iv) recorded response times of less than 0.3 seconds on more than 10% of the Implicit Association Test (IAT), as these responses were deemed unreliable [18].

This study was approved by the Ethics Committee of the University of Tokyo and conducted in accordance with the principles of the Declaration of Helsinki. It also adhered to the Strengthening the Reporting of Observational Studies in Epidemiology (STROBE) guidelines.

### Measures

Participants completed an anonymous questionnaire assessing their turnover intention, WFC, implicit gender bias, sociodemographic characteristics, and work-related and personal details. The survey was conducted anonymously to encourage candid responses and minimize the potential for social desirability bias. Sociodemographic characteristics included gender, age, place of birth, and university. Work-related factors included working conditions, job position, board certification in ophthalmology, possession of a doctoral degree, perceived job fit, and perceptions on whether women are active in the department. Personal details included marital status, spouse’s occupation and years of employment, number of children, age of the youngest child, and hours spent per day on housework, childcare, and caregiving (Online Resource 1).

The primary outcome was turnover intention, assessed using the Intention to Leave Scale, which has demonstrated reliability and validity [19]. This study employed the Japanese version [20] which has been used in previous research [20, 21]. The scale comprises four items [20], with participants rating their feelings about leaving their organization over the past month on a 5-point Likert scale (e.g., ‘I consider my decision to work for this employer as an obvious mistake’) (Online Resource 1). Higher scores indicate greater turnover intention [19, 20]. Consistent with previous studies [19–21], this variable was treated as continuous in the analysis. We measured WFC using the 18-item Japanese version of the Work–Family Conflict Scale [12, 22], developed based on the multidimensional Work–Family Conflict Scale [23]. This scale assesses two distinct dimensions: work interference with family (WIF) and family interference with work (FIW) [6, 12, 23] (Online Resource 1).

A substantial body of work has documented that WIF and FIW are theoretically distinct, influenced by different factors, and resulting in different outcomes [24]. These two forms of conflict are not interchangeable and often correlate differently with other variables [12, 25]. Responses for each subset were averaged, with higher scores indicating greater WFC [12]. The IAT quantifies the direction and strength of implicit biases and is characterized by high internal consistency and predictive validity [18, 26]. The Gender–Career IAT used in this study assesses implicit associations between two pairs of concepts: men–career and women–family (stereotypical pairings) versus men–family and women–career (non-stereotypical pairings) [27]. The IAT is based on the logical premise that easier pairings (faster responses, fewer errors) reflect more significant implicit associations than more difficult pairings (slower responses, more errors) [26, 27]. Differences in response times between stereotypical and non-stereotypical pairings were standardized by dividing them by the standard deviation (SD) of all response latencies. The IAT D score, an effect size measure ranging from −2 to +2, was used. Higher positive values indicate a stronger bias toward stereotypical pairings [27] (Online Resource 1).

### Statistical analysis

First, we described the characteristics of the participants. Second, we reported the means and SDs for turnover intention, WIF, FIW, and D scores. Medians and interquartile ranges (IQR) were reported for job fit. All analyses were conducted separately by gender and hospital type. We assessed the internal consistency of turnover intention, WIF, and FIW measures using Cronbach’s α. IAT D scores were analyzed using a one-sample *t*-test to determine whether the mean D score significantly differed from zero. We used *t*-tests to compare continuous variables between two independent groups with normal distributions. Mann–Whitney U tests were used for non-normally distributed variables. We conducted confirmatory factor analysis (CFA) based on the factor structure of the Japanese version of the WFC scale to examine whether the factor structure fit our observed data. The criteria for model fit were set at Root Mean Square Error of Approximation (RMSEA) ≤ 0.05 and Comparative Fit Index (CFI) ≥ 0.95. CFA evaluates the construct validity of a theoretical model by testing how well the observed data fit a hypothesized factor structure based on prior theory [28].

Third, we used Spearman’s rank correlation coefficient to examine correlations between variables. Fourth, multivariable regression analysis was conducted to evaluate the associations between WIF, FIW, and D scores and turnover intention. The following covariates were selected based on their previously reported associations with turnover intention: age [5, 9, 29, 30], gender [29, 30], job fit [31], job position [5] clinical work hours per week [29] single status [29], and childcare responsibilities [30]. Additionally, variables that showed significant correlations with turnover intention were adjusted for in the regression analysis. Given the limited sample size, we restricted the number of adjustment variables.

Fifth, to examine whether the associations between WIF, FIW, and D scores and turnover intention varied by physician age, we included an interaction term between each exposure variable (WIF, FIW, or D Score) and an age indicator (≥ 45 years old vs. < 45 years) in the model. Continuous variables were mean-centered before creating interaction terms to reduce multicollinearity. Due to the small sample size, each exposure variable was analyzed separately. That is, when the interaction between one exposure variable and age was included, interactions between other exposure variables and age were excluded. The threshold of 45 years was determined based on previous research indicating that fathers aged 30–44 were more inclined to balance work and family life [32]. Furthermore, in Japan, those aged 30–44 have experienced societal changes such as the enactment of the Act on Advancement of Measures to Support Raising Next-Generation Children and the introduction of coeducational home economics classes [33]. To examine whether these generational experiences are associated with turnover intention, WFC, and implicit gender bias, we tested the interaction using Wald’s test.

All *p*-values were two-sided, with statistical significance set at *p* <.05. We assessed multicollinearity using Variance Inflation Factors(VIFs), and a values above five indicated multicollinearity.

All statistical analyses were performed using SPSS and SPSS Amos, version 29.0. Data analysis was performed from February 2024 to May 2025. Patients and the public were not involved in the design, conduct, reporting, or dissemination of this study.

## Results

### Participant characteristics and item scores

We sent a web-based questionnaire to 128 potential participants (men: 68, women: 60) among the 141 ophthalmologists working full-time at university-affiliated or general hospitals in the Kanto region, whose email addresses were available. We received responses from 89 participants. We excluded one participant who did not provide consent and eight who did not complete the questionnaire, resulting in 80 valid responses, a response rate of 62.5% (80/128).

Among those who completed all questions, we excluded three participants registered as full-time physicians but working fewer than 32 hours per week in a hospital setting and three whose IAT reaction times were under 300 milliseconds in more than 10% of trials. Thus, the final analysis included 74 ophthalmologists, yielding an effective response rate of 57.8%.

The study participants included 36 male and 38 female ophthalmologists, with a mean (SD) age of 41.5 (10.8) years. Of them, 25 (33.8%) were working at university-affiliated hospitals, and 49 (66.2%) at general hospitals. The number of night duties per month was significantly higher among the university-affiliated hospital ophthalmologists than the general hospital ophthalmologists (*p* < 0.001). There was no significant difference in job position distribution by gender. Among the 74 participants, 29 held management positions, of which 14 were occupied by men and 15 by women. Twenty-five were staff physicians (13 men, 12 women), and 20 were non-board-certified ophthalmologists (9 men, 11 women). The difference in job position by gender was not statistically significant (*p* = 0.27). In terms of academic qualifications, 35 participants held a PhD degree (18 men, 17 women), with no significant gender difference (*p* = 0.65). Six physicians worked more than 60 hours per week (Table 1). None of the men or women in their 20s had spouses who were homemakers. No participants in their 20s had children, while among those in their 30s with children, 92.3% had children aged six or younger. Only those in their 50s and 60s had prior caregiving experience. Regarding perceptions on whether women are active in the department, 82.4% of participants agreed or somewhat agreed with the statement (Online Resource 1).

**Table 1:** Sociodemographic characteristics of participating hospital ophthalmologists

	Number of participants (N=74)	*n* (%)
*Gender*	Men	36 (48.6)
	Women	38 (51.4)
*Age*	26–29	12 (16.2)
	30–39	24 (32.4)
	40–49	20 (27.0)
	50–66	18 (24.3)
*Position*	Fellow	20 (27.0)
	Staff physician	25 (33.8)
	Management position	29 (39.2)
*Board-certified doctor of ophthalmology*	Yes	55 (74.3)
	No	19 (25.7)
*PhD*	Yes	35 (47.3)
	No	39 (52.7)
*Number of ophthalmologists at the hospital*	1	2 (2.7)
	2–5	21 (28.4)
	6–10	26 (35.1)
	11–20	7 (9.5)
	More than 21	18 (24.3)
*Number of hospital beds*	20–199	1 (1.4)
	200–399	14 (18.9)
	400–599	24 (32.4)
	600–799	13 (17.6)
	More than 800	22 (29.7)
*Clinical work hours per week*	32h–40h	15 (20.3)
	40h–50h	36 (48.6)
	50h–60h	17 (23.0)
	More than 60h	6 (8.1)
*Median number of nights on duty per month*	0	33 (44.6)
	1	21 (28.4)
	2	14 (18.9)
	3	5 (6.8)
	4	1 (1.4)
*Place of birth*	Kanto	44 (59.45)
	Other	30 (40.5)
*University*	Urban public university	29 (39.2)
	Urban private university	17 (22.9)
	Rural public university	25 (33.8)
	Rural private university	3 (4.05)
*Did you choose to major in medicine because of your interest in medicine and healthcare? (Reason for higher education)*	I disagree completely	1 (1.4)
	Somewhat disagree	3 (4.1)
	Neither	5 (6.8)
	Somewhat agree	30 (40.5)
	I agree completely	35 (47.3)
*Marital status*	Married	54 (73.0)
	Unmarried	16 (21.6)
	No response	4 (5.4)
*Spouse’s occupation*	Full-time physician	29 (53.7)
	Part-time physician	4 (7.4)
	Full-time non-physician healthcare professional	0
	Part-time non-physician healthcare professional	4 (7.4)
	Full-time or self-employed non-healthcare professional	9 (16.7)
	Part-time non-healthcare professional	1 (1.4)
	Homemaker	7 (13.0)
*Spouse’s years of employment*	Less than 1 year	2 (3.7)
	Over 1 year and under 5 years	14 (25.9)
	Over 5 years and under 10 years	9 (16.7)
	Over 10 years and under 20 years	13 (24.1)
	Over 20 years and under 30 years	11 (20.4)
	Over 30 years	5 (9.3)
*Number of children*	None	28 (37.8)
	1	17 (23.0)
	2	20 (27.0)
	3	5 (6.8)
	More than 4	4 (5.4)
*Youngest child’s age*	0–3	9 (19.6)
	4–7	12 (26.1)
	8–11	5 (10.9)
	More than 12	20 (43.5)
*Caregiving experience*	Yes	5 (6.8)
	No	66 (89.2)
	No response	3 (4.1)
*Time spent per day on housework, childcare, and caregiving*	Less than 1 hour	29 (39.2)
	More than 1 hour and less than 3 hours	25 (33.8)
	More than 3 hours and less than 5 hours	14 (18.9)
	More than 5 hours and less than 7 hours	5 (6.8)
	More than 7 hours	1 (1.4)

Note: Management positions include professors, associate professors, and senior lecturers at university hospitals, as well as department heads or chief physicians at general hospitals.

Cronbach’s α was used to evaluate scale reliability. Turnover intention demonstrated acceptable reliability (α = 0.76), while both WIF (α = 0.86) and FIW (α = 0.85) showed good reliability. A one-sample *t*-test of D scores revealed a significant difference from zero (*t*(73) = 8.713; 95% confidence interval [CI], 0.28–0.44; *p* < 0.001). The median turnover intention score was 2.50 (IQR: 2.00–3.00). Paired *t*-tests showed that both men and women had higher WIF than FIW (men: *p* < 0.001; women: *p* < 0.001). No significant gender differences were observed in turnover intention, WIF, FIW, or D scores (Table 2). Additionally, no significant differences in these scores were found across age groups (20s, 30s, 40s, and 50s–60s). Furthermore, no significant differences were observed in turnover intention (*p* = 0.65), WIF (*p* = 0.52), or FIW (*p* = 0.46) between university-affiliated hospital and general hospital ophthalmologists. The initial CFA model did not meet acceptable fit criteria (RMSEA = 0.21, 90% CI [0.20, 0.23]; CFI = 0.54). When error covariances were added to account for correlations between items within subdomains, model fit improved (RMSEA = 0.11, 90% CI [0.09, 0.14]; CFI = 0.89). Although the model fit was suboptimal, we used the scale as is in this study, given that its reliability and validity have been confirmed in Japanese populations. The limitations associated with this are discussed later.

**Table 2:** Item scores by gender

	Men	Women	*p*
Turnover intention [SD]	2.58 [0.87]	2.63 [0.65]	0.78
Work interference with family (WIF)^a^ [SD]	2.97 [0.88]	3.07 [0.65]	0.59
Family interference with work (FIW)^a^ [SD]	2.54 [0.81]	2.36 [0.53]	0.26
D score (gender-career IAT)^a^ [SD]	0.28 [0.34]	0.43 [0.35]	0.07
Job fit^b^ (IQR)	4.00 (4.00-4.75)	4.00 (4.00-5.00)	0.74

Note: SD, standard deviation; IQR, interquartile range

^a^ *p*-values were calculated using the *t*-test; ^b^ *p*-values were calculated using the Mann–Whitney U test.

WIF and FIW were calculated as the sum of all respective items divided by 9.

### Correlation and regression analyses

We examined correlations between turnover intention and other variables. WIF was positively associated with turnover intention (ρ = .29, *p* = 0.012), while job fit (ρ = .45, *p* < 0.001) and job position (ρ = .31, *p* = 0.007) were negatively associated with turnover intention, indicating that higher job positions corresponded to lower turnover intention. No significant associations were observed with other variables, such as clinical work hours per week, youngest child’s age, or D scores (Table 3).

**Table 3:** Spearman correlation test for various factors

	(1)	(2)	(3)	(4)	(5)	(6)	(7)	(8)	(9)	(10)	(11)	(12)	(13)	(14)	(15)	(16)	(17)
(1) Turnover intention																	
(2) Work interference with family (WIF)	.29*																
(3) Family interference with work (FIW)	.21	.56**															
(4) D score (Implicit gender-career bias)	− .14	− .045	− .18														
(5) Job fit	− .45**	− .29*	− .24*	.11													
(6) Men dummy	− .064	− .018	.060	− .20	− .038												
(7) Age	− .21	.12	.19	− .11	.13	.083											
(8) Job position	− .31**	.15	.16	− .052	.16	.018	.85**										
(9) Number of ophthalmologists at the hospital	.064	.13	.071	.10	.014	.18	− .27*	− .25*									
(10) Number of hospital beds	.058	.17	.11	− .058	.056	.33**	− .028	− .031	.68**								
(11) Clinical work hours per week	.14	.29*	− .090	.080	− .23	.20	− .066	.002	.29*	.31**							
(12) Median number of nights on duty per month	.078	.15	.17	.009	− .057	.23	− .36**	− .32**	.54^**^	.37**	.23*						
(13) Reasons for higher education	− .17	.021	− .16	.16	.62**	− .15	.18	.20	− .091	.025	− .065	− .13					
(14) Married dummy	− .11	.063	.042	.093	.002	.17	.39**	.36**	− .10	− .10	− .24*	− .20	− .037				
(15) Spouse’s years of service	− .11	− .040	.23	.12	.15	− .46**	.47**	.56**	− .17	− .27*	.04	− .37**	.086				
(16) Number of children	− .12	.032	.10	.045	.14	.18	.62**	.61**	− .19	− .001	− .20	− .37**	.14	.59**	.14		
(17) Youngest child’s age	− .072	.19	.041	− .067	.057	.054	.83**	.50**	− .17	.04	.31*	− .047	.16	− .062	.22	.077	
(18) Time spent per day on housework, childcare, and caregiving	.060	− .049	.089	.15	.17	− .28*	.21	.27*	− .012	− .07	− .16	− .29^*^	.15	.30**	.37**	.44**	− .40**

Note: Pairwise analysis. *p < 0.05; **p < 0.01; ***p < 0.001

We found a significant association between WIF and turnover intention (adjusted difference: 0.30; 95% CI, 0.075–0.53; *p* = 0.010). However, FIW (adjusted difference, 0.099; 95% CI, 0.16 to 0.36; *p* = 0.45) and the D score (adjusted difference, 0.25; 95% CI, 0.66 to 0.16; *p* = 0.23) were not significantly associated with turnover intention (Table 4). All VIFs were below 2.0.

**Table 4:** Multivariable regression analysis with turnover intention as the dependent variable

Independent variables	B	95% CI	Standardized β	*p*
Work interference with family (WIF)	0.30	(0.075–0.53)	0.31	0.010**
Family interference with work (FIW)	0.099	(− 0.16 to 0.36)	0.089	0.45
D score (Implicit gender bias)	-0.25	(− 0.66 to 0.16)	− 0.11	0.23
Job fit	-0.33	(− 0.52 to − 0.14)	− 0.34	0.001**
Management dummy	-0.53	(− 0.89 to − 0.17)	− 0.34	0.005**
Staff physicians dummy	-0.14	(− 0.52 to 0.24)	− 0.090	0.45

Note: CI: confidence interval. Management positions include professors, associate professors, and senior lecturers at university hospitals, as well as department heads or chief physicians at general hospitals. Standardized β coefficients are reported for comparison.

Pairwise analysis. *p < 0.05; ** p < 0.01; *** p < 0.001.

We additionally conducted subgroup multiple regression analyses by workplace setting. Among general hospital physicians, turnover intention was significantly associated with WIF (adjusted difference, 0.38; 95% CI, 0.10–0.65; *p* = 0.009), job fit (adjusted difference, 0.41; 95% CI, 0.63 to 0.18; *p* < 0.001), and management position (adjusted difference, 0.57; 95% CI, 1.00 to 0.13; *p* = 0.013). In contrast, no significant associations were found among university-affiliated physicians. All VIFs were below 2.5.

### Interaction effects on turnover intention

In Model 1, we examined the interaction between WIF and being 45 years or older (Table 5). The results indicate that the association between WIF and turnover intention varied by age (*p*-value for interaction = 0.03). The coefficient for WIF was 0.46 (95% CI 0.20–0.72), while the interaction term had a coefficient of 0.42 (95% CI 0.80 to 0.050), indicating that WIF was associated with turnover intention only among physicians younger than 45 years.

**Table 5:** Interaction effects of WIF, FIW, and D score with age (≥ 45 years) on turnover intention

Independent variables	Model 1		Model 2		Model 3	
	B	95% CI	B	95% CI	B	95% CI
Work interference with family (WIF)	0.46	(0.20–0.72)	0.32	(0.10–0.55)	0.31	(0.076–0.54)
Family interference with work (FIW)	0.088	(− 0.17 to 0.34)	0.25	(− 0.042 to 0.53)	0.093	(− 0.17 to 0.36)
D score (implicit gender bias)	− 0.23	(− 0.63 to 0.17)	− 0.23	(− 0.63 to 0.17)	− 0.35	(− 0.92 to 0.21)
Aged 45 years or older dummy	0.15	(− 0.32 to 0.62)	0.18	(− 0.29 to 0.65)	0.11	(− 0.38 to 0.59)
WIF × 45 years or older dummy	− 0.42	(− 0.80to − 0.050)				
FIW × 45 years or older dummy			− 0.51	(− 0.95 to − 0.065)		
D Score × 45 years or older dummy					0.25	(− 0.58 to 1.08)

Note: All models are multiple regression models adjusted for job fit, management dummy, and staff physician dummy variables. WIF, FIW, and D scores were treated as exposure variables. Continuous variables were mean-centered before creating interaction terms to reduce multicollinearity

Model 1 examined the interaction between WIF and being 45 years or older (vs. younger than 45)

Model 2 examined the interaction between FIW and being 45 years or older (vs. younger than 45)

Model 3 examined the interaction between the D score and being 45 years or older (vs. younger than 45)

*CI* confidence interval

In Model 2, we tested the interaction between FIW and being 45 years or older. The results similarly suggest that the association between FIW and turnover intention varied by age (*p*-value for interaction = 0.03). The coefficient for FIW was 0.25 (95% CI 0.042 to 0.53), while the interaction term had a coefficient of 0.51 (95%CI 0.95 to 0.065), indicating that the association between FIW and turnover intention differed by age. The association between the D score and turnover intention did not vary by physician age. All VIFs were below 4.0.

## Discussion

The findings of this cross-sectional study suggest that turnover intention is positively associated with WIF but not with FIW or implicit gender bias. To the best of our knowledge, this is the first study to examine the associations between WIF, FIW, implicit gender bias, and turnover intention among Japanese hospital ophthalmologists.

A meta-analytical review has established that WFC is a key predictor of turnover intention [11]. Specifically, high WIF has been associated with a greater turnover intention, with previous research identifying both turnover intention and high turnover rates as consequences of work interfering with private life [10, 24]. In contrast, FIW has not been associated with turnover intention in previous studies [12]. Consistent with these findings, our study found that WIF correlated with turnover intention, whereas FIW did not. This may be explained by the greater permeability of family boundaries compared to work boundaries, making WIF more prevalent [10]. Additionally, individuals typically have less control over work-related time demands than family-related time demands [34], which may contribute to higher WIF but lower FIW [35]. Previous studies indicate that psychological factors such as work stress and job satisfaction may mediate the relationship between WIF and turnover intention [36, 37]. Although these mediators were not assessed in the present study, future research should explore these associations.

Meta-analyses indicate that men and women often experience similar levels of WIF and FIW [25]. However, when placed in identical work–family situations, their levels of WFC may differ depending on their implicit gender stereotypes. Implicit gender biases significantly influence individuals’ behaviors. For example, mothers with more traditional internalized stereotypes tend to experience greater guilt and are more likely to perceive work–family situations as conflicts, compared to fathers or more egalitarian mothers [13]. Nonetheless, an age-cohort study has shown that both men and women increasingly seek to move away from traditional gender roles. In particular, Generation Y (30–44 years old at the time of this study) includes a new generation of fathers who aspire to spend more time with their families and seek realistic ways to balance work and family life [32].

Despite this shift, gender role biases remain deeply ingrained in Japanese society [14, 15]. Japanese men, in particular, have been criticized for their limited participation in housework and childcare [30]. In our study, a one-sample *t*-test of D scores revealed an implicit gender bias associating men with careers and women with family roles. However, although such biases were present in both men and women, we found no significant gender differences in time spent on work, housework, and childcare among participants in their 20s and 30s (Online Resource 1). In addition, the lack of significant gender differences in WIF and FIW suggests that perceptions of WFC may be evolving among younger individuals. The workplace environment was reported to be an essential factor influencing WFC [12, 13, 25]. Among the participants, no significant gender differences were observed across job positions. Additionally, 82.4% of participants perceived the department as an organization where women are active. These factors may also explain why men and women ophthalmologists in this study reported similar levels of WFC.

Meta-analyses suggest that the more time an individual spends in a role—or the more they specialize or become involved in it—the greater the perceived interference with a secondary role [25]. Employees with higher job involvement, workload, or job stress, as well as those who spend more time at work, tend to experience more WIF than FIW [12, 13, 25]. Conversely, employees who find fulfillment in their work report lower WIF [38]. In contrast, FIW has been associated with family stress, younger children, and a larger number of children [6]. Furthermore, studies have found a direct relationship between WIF and FIW, indicating that an increase in one tends to correspond with an increase in the other [39].

In this study, we observed a positive correlation between WIF and FIW, as well as between WIF and weekly clinical work hours. Additionally, a negative correlation was found between job fit and both WIF and FIW. However, no correlation was found between FIW and factors such as the age of the youngest child or the number of children, which are typically considered antecedents of FIW. Previous studies have reported that FIW is higher among individuals with more children [6]. However, in our study, the majority of participants had no children, and only a small percentage had three or more children, which may explain the absence of this expected association.

Consistent with previous studies, this study found that WIF was correlated with turnover intention, and working hours were identified as a workplace factor contributing to WIF. In Japan, in addition to providing patient care, university-affiliated hospitals play an important role as institutions for medical education and advanced research [40], which may create structural differences in the work responsibilities of ophthalmologists compared to those in general hospitals. Although this study demonstrates that night duties were more frequent among university-affiliated hospital physicians, no significant differences were found in WIF, FIW, and turnover intention between the two groups. Among general hospital physicians, turnover intention was significantly associated with WIF, job fit, and management position. In contrast, no significant associations were found among university-affiliated physicians, possibly due to the smaller sample size. These findings indicate that clinical workload and organizational roles may influence turnover intention more strongly in general hospital settings. Previous studies indicate that factors reducing WIF include shorter working hours [12, 25], flexible work schedules [41], supervisor support [12, 25], and targeted training for employees experiencing high WFC [42]. The interaction analysis in this study indicates that WIF was more significantly associated with turnover intention among individuals younger than 45 years compared to those aged 45 or older.

Men and women in their 30s are also more likely to take on increased family responsibilities, such as childbirth and child-rearing [43]. In Japan, the average age of mothers at the birth of their first child is 30.9 years [44], and WFC has been shown to be particularly prevalent in age groups that overlap with childbearing and childcare. A German study found that young hospital physicians were more likely to have young children and, consequently, experienced high family and parenting demands, leading to high levels of WFC [9]. In Japan, work-style reforms for physicians were implemented in 2024 to reduce working hours. Although the Ministry of Health, Labor and Welfare mandates top management training, we recommend that hospital managers and administrators recognize that work and family responsibilities are not mutually exclusive [45]. Training programs should be implemented for physicians with high WIF, particularly those aged 30–44, regardless of gender. Furthermore, equipping managers with the skills to support their subordinates in balancing work and family life is essential.

This study has some limitations. First, as a cross-sectional study, it does not allow for the examination of causal relationships between turnover intention, WIF, FIW, and various characteristics. However, we selected these outcomes and predictors based on previous qualitative studies and literature from psychological, sociological, and economic perspectives. Future studies should employ a longitudinal design. Second, our sample size was small and regionally limited, which should be addressed in future investigations with larger, more diverse samples. In particular, study participants were limited to physicians in urban and university-affiliated hospitals and general hospitals in the Kanto region. Cultural and institutional differences in rural and/or non-university-affiliated settings may affect the generalizability of the findings. An exploratory study of rural physicians reports that factors such as understaffing, heavy workloads, and a lack of career opportunities for partners contributed to physicians’ turnover [46]. Additionally, a study conducted in the United States indicates that ophthalmologists working at academic institutions or in rural areas may be more likely to leave their positions [47]. These factors indicate that regional contexts may shape turnover intention differently. Further, this study did not directly measure the educational and research responsibilities specific to university-affiliated hospitals, which may have affected WFC and turnover intention. Future research should clarify whether and how such cultural and institutional roles shape these outcomes. Third, although we examined whether job fit differed by board certification status or academic/managerial position, no significant differences were obtained. However, considering the limited sample size, future research should further investigate how career-stage milestones may influence job fit and turnover intention among ophthalmologists. Fourth, although income levels are known to influence physician turnover [48] and are associated with WFC [12], this study did not include any financial questions due to concerns regarding participant burden and the sensitivity of personal information. Future research should address how financial factors contribute to turnover intention in this context. Fifth, this study did not collect specific information on whether the participating hospitals provided flexible work schedules and on-site childcare facilities, nor did it assess the extent to which these policies were utilized. While most participants perceived the department as an organization where women are active, the relationship between this perception and WFC remains unclear due to a lack of prior research. Sixth, this study did not include individuals who had actually left their jobs. Although the turnover intention may change over time, a review and meta-analysis of longitudinal studies have shown that turnover intention is the most significant predictor of actual turnover [19]. Additionally, although this scale demonstrated validity and was used in previous studies, its criterion validity among Japanese physicians remains unconfirmed due to a lack of longitudinal research. Seventh, although the model fit indices from the CFA were suboptimal for the WFC scale, each subscale showed high reliability, and previous Japanese studies have confirmed its validity. Therefore, we used the Japanese version of the WFC scale. However, the suboptimal CFA results in this study warrant caution in interpreting the findings, as some items within the WIF and FIW subscales may potentially be interrelated. Furthermore, although the survey was conducted anonymously to help minimize social desirability bias, this potential bias cannot be completely ruled out.

In conclusion, this cross-sectional study found a positive association between WIF and turnover intention among Japanese hospital ophthalmologists, even after accounting for implicit gender bias. In future studies, we aim to investigate whether WIF and turnover intention can be mitigated by reducing working hours, offering flexible work arrangements, training supervisors to support better work–life balance, and providing targeted training for physicians experiencing high levels of WIF.

## Supplementary Information

Below is the link to the electronic supplementary material.

Supplementary file1 (DOCX 56 KB)

## Data Availability

Data are available upon reasonable request. The data supporting this study’s findings are available on request from the corresponding author. Access may be granted to bona fide researchers under a data-sharing agreement. The data are not publicly available due to ethical restrictions.
