# Supplementary material for: Association of work-family conflict with turnover intention among hospital ophthalmologists in Japan: a cross-sectional study
Source: Jpn J Ophthalmol. 2025 Sep 8;70(2):394–405. doi: 10.1007/s10384-025-01275-3 (PMC13091806; doi:10.1007/s10384-025-01275-3)
Supplement: Supplementary file 1 — Supplementary file1 (DOCX 56 KB) [file 10384_2025_1275_MOESM1_ESM.docx]

**Online Resource 1**

*Survey Questions*

*Turnover intention*

Turnover intention was measured using a four-item scale [1, 2].

1. I consider my decision to work for this employer an obvious mistake.

2. If it had been easier to change employers, I would have quit long ago.

3. I am equally willing to work for another employer.

4. Before I change employers, a lot has to happen. (This item is reverse-coded)

The English version of the Intention to Leave Scale was translated into Japanese and modified for clarity using plain Japanese expressions. In the Japanese version, respondents used a 5-point Likert scale to indicate the extent to which they had considered leaving their organization in the past month. The Intention to Leave Scale was scored with response options for items 1–3: 1=5, 2=4, 3=3, 4=2, and 5=1. Item 4, which is reverse-coded, was scored as 1=1, 2=2, 3=3, 4=4, and 5=5. The mean turnover intention score was calculated by dividing the total score by 4. Participants responded to all four questions using the following 5-point Likert scale:

1. Strongly agree

2. Agree

3. Neither agree nor disagree

4. Disagree

5. Strongly disagree completely

*Work–family conflict*

We used the 18-item Japanese version of the Work–Family Conflict Scale [3, 4].

*Work interference with family (WIF)*

1. My work keeps me from my family activities more than I would like.

2. The time I must devote to my job keeps me from participating equally in household responsibilities and activities.

3. I have to miss family activities due to the amount of time I must spend on work responsibilities.

4. When I get home from work, I am often too frazzled to participate in family activities/responsibilities.

5. I am often so emotionally drained when I get home from work that it prevents me from
contributing to my family.

6. Due to all the pressures at work, sometimes when I come home, I am too stressed to do the
things I enjoy

7. The problem-solving behaviors I use in my job are not effective in resolving problems at home.

8. Behavior that is effective and necessary for meat work would be counterproductive at home.

9. The behaviors I perform that make me effective at work do not help me to be a better parent and spouse.

*Family interference with work (FIW)*

10. The time I spend on family responsibilities often interferes with my work responsibilities.

11. The time I spend with my family often causes me not to spend time in activities at work that could be helpful to my career.

12. I have to miss work activities due to the amount of time I must spend on family responsibilities.

13. Due to stress at home, I am often preoccupied with family matters at work.

14. Because I am often stressed from family responsibilities, I have a hard time concentrating on my work.

15. Tension and anxiety from my family life often weaken my ability to do my job.

16. The behaviors that work for me at home do not seem to be effective at work.

17. Behavior that is effective and necessary for me at home would be counterproductive at work.

18. The problem-solving behavior that works for me at home does not seem to be as useful at work.

Participants responded using a 5-point Likert scale ranging from 1 (strongly disagree) to 5 (strongly agree):

1. Strongly disagree

2. Disagree

3. Neither agree nor disagree

4. Agree

5. Strongly agree

*Implicit gender-career bias*

We conducted a gender–career Implicit Association Test (IAT). Participants were instructed to quickly categorize Japanese words that appeared on a computer screen (referred to as stimuli) into different categories. The stimuli included male-related words (husband, boy, man, father, uncle), female-related words (wife, girl, woman, mother, aunt), career-related words (salary, management, job, profession, promotion), and family-related words (marriage, family, home, child, relatives) [5]. A scoring algorithm was used to calculate IAT D scores for each participant [6]. The IAT was implemented online using Psychexp [7] which allows the test to be run on any platform with a modern web browser. Participants received their results after completing the survey. The Psychexp IAT includes a built-in procedure requiring participants to provide a correct response after making an incorrect one before proceeding to the next trial. Response time, measured from the start of the stimulus presentation to the correct response, was recorded.

*Job fit*

We used an item from the Job Compatibility Check manual, designed for robust stress assessment using the Brief Job Stress Questionnaire from the Ministry of Health, Labour, and Welfare. Higher scores indicated a better job fit [8].

Survey item:

The job description fits me.

1. Strongly disagree.

2. Disagree.

3. Neither agree nor disagree

4. Agree.

5. Strongly agree.

*Demographic information*

1. What is your gender?

a. Man

  b. Woman

c. Other

2. What is your age?

3. Where were you born?

a. Hokkaido

b. Tohoku

c. Kanto

d. Chubu

e. Kinki

f. Chugoku

g. Shikoku

h. Kyushu & Okinawa

i. Other (outside of Japan)

4. What type of university did you attend?

a. National/public university in one of the five major metropolitan areas of Japan (Sapporo, Tokyo, Nagoya, Osaka, and Fukuoka)

b. Private university in one of the five major metropolitan areas

c. National/public university in a region outside the five major metropolitan areas

d. Private university in a region outside the five major metropolitan areas

5. Are you a board-certified ophthalmologist?

a. Yes

b. No

6. Do you have a doctoral degree?

a. Yes

b. No

7. What is your current job position?

a. Lecturer or higher at a university-affiliated hospital

b. Assistant Professor or Specially Appointed Lecturer at a university-affiliated hospital

c. Staff Physician at a university-affiliated hospital (Board-certified ophthalmologist)

d. Resident at a university-affiliated hospital (ophthalmology specialist training program)

e. Other full-time physician at a university-affiliated hospital (Completed ophthalmology specialist training program but not board-certified)

f. Department Head or higher at a general hospital

g. Chief Physician at a general hospital

h. Staff Physician at a general hospital (Board-certified ophthalmologist)

i. Resident at a general hospital (ophthalmology specialist training program)

j. Other full-time physician at a general hospital (completed ophthalmology specialist training program but not board-certified)

k. Part-time physician

l. Other

8. How many full-time ophthalmologists work at your hospital?

a. 1

b. 2

c. 3

d. 4

e. 5

f. 6

g. 7

h. 8

i. 9

j. 10

k. 11–15

l. 16–20

m. 21–25

n. 26–30

o. More than 31

9. What is the bed capacity of your hospital?

a. 20–199 beds

b. 200–399 beds

c. 400–599 beds

d. 600–799 beds

e. More than 800 beds

10. What were your average weekly working hours (including overtime and on-call hours) over the past month?

a. 32 to less than 40 hours

b. 40 to less than 45 hours

c. 45 to less than 50 hours

d. 50 to less than 55 hours

e. 55 to less than 60 hours

f. More than 60 hours

11. How many nights have you worked in the past month (excluding on-call shifts)?

a. None

b. Once

c. Twice

d. Three times

e. Four times

f. Five times

g. Six times

h. Seven times

i. More than Eight times

12. Are you married?

a. Yes

b. No

c. Prefer not to answer

13. What is your spouse's occupation?

a. Full-time physician

b. Part-time physician

c. Full-time non-physician healthcare professional

d. Part-time non-physician healthcare professional

e. Full-time or self-employed non-healthcare professional

f. Part-time non-healthcare professional

g. Homemaker

14. What is your spouse’s total years of continuous employment?

*(If they have changed jobs, please include the total number of years across all jobs. Periods of maternity, childcare, and caregiver leave should be included in the total).*

a. Less than 1 year

b. More than 1 year and under 5 years

c. More than 5 years and under 10 years

d. More than 10 years and under 20 years

e. More than 20 years and under 30 years

f. More than 30 years

15. How many children do you have?

a. None

b. One

c. Two

d. Three

e. More than four

16. What is the age of your youngest child?

a. 0 years

b. 1 year

c. 2 years

d. 3 years

e. 4 years

f. 5 years

g. 6 years

h. 7 years

i. 8 years

j. 9 years

k. 10 years

l. 11 years

m. 12 years

n. 13 years or older

17. Do you currently provide caregiving?

a. Yes, I currently provide care

b. No, I do not currently provide care

c. Prefer not to answer

18. What is your average daily time spent on housework, childcare, and caregiving?

a. Less than 1 hour

b. More than 1 hour but less than 3 hours

c. More than 3 hours but less than 5 hours

d. More than 5 hours but less than 7 hours

e. More than 7 hours

19. Is the Department of Ophthalmology of the University of Tokyo an organization where

women are active?

a. I agree completely

b. I agree

c. Neither agree nor disagree

d. I disagree

e. I disagree completely

*Clinical work time per week and hours spent on housework, childcare, and caregiving per day by gender, age, and hospital type.*

We analyzed the clinical work time per week and hours spent on housework, childcare, and caregiving per day by gender, age groups and hospital type. Previous studies reported that turnover intention was associated with clinical work time [9, 10] and childcare [11]. Meta-analyses showed that working hours were related to WIF, while time spent with family, such as on housework and childcare, was related to FIW [12]. We used the Mann–Whitney U test to compare gender, age and hospital type differences.

*Job fit score comparison by career-stage indicators*

To examine whether job fit scores differed by career-stage milestones, we compared job fit between ophthalmologists with and without board certification in ophthalmology, and between those holding academic or managerial positions (senior lecturer or higher) and others. The Mann–Whitney U test was used for these comparisons.

**Supplementary Results**

*Clinical work time per week and time spent on housework, childcare, and caregiving per day by gender, age, and hospital type.*

We analyzed weekly clinical work hours and daily time spent on housework, childcare, and caregiving across four age groups, stratified by gender. No statistically significant difference was observed between men and women in terms of clinical work hours per week (*p* = 0.080), although men tended to work longer hours. By contrast, women spent significantly more time on housework, childcare, and caregiving per day (*p* = 0.018).

In addition, no significant gender differences in clinical work hours were found within each age group (20s, *p*=0.096; 30s, *p*=0.15; 40s, *p*= 0.11; 50s, *p*= 0.89). However, women in their 40s to 60s spent significantly more time on housework, childcare, and caregiving than men (40s: *p*=0.009, 50s: *p*=0.025). No significant gender differences were found among individuals in their 20s and 30s (20s: *p*=0.075, 30s: *p*=0.67). We also compared weekly clinical work hours and daily time spent on housework and childcare between university-affiliated hospital physicians and general hospital physicians. Similarly, no significant differences were found in clinical work hours per week (*p* = 0.098) and daily time spent on housework and childcare (*p* = 0.77) between physicians working at university-affiliated hospitals and those at general hospitals.

*Job fit score comparison by career-stage indicators*

There were no significant differences in job fit scores between ophthalmologists with and without board certification of ophthalmology (*p* = 0.70), or between those in academic and/or management positions and those in other positions (*p* = 0.11).

*Perceptions of women’s active participation in the department*

Participants were asked whether they perceived the Department of Ophthalmology of the University of Tokyo as an organization where women are active. Among the participants, 18.9% (n = 14) answered “I agree completely,” 63.5% (n = 47) answered “Somewhat agree,” 13.5% (n = 10) answered “Neither agree nor disagree,” and 4.1% (n = 3) answered “Somewhat disagree.” No participants selected “I disagree completely.”

**Supplementary References**

1. Geurts S, Schaufeli W, De Jonge J. Burnout and intention to leave among mental health-care professionals: a social psychological approach. *J Soc Clin Psychol*. 1998;17(3):341–362.

2. Tsuno K, Kawachi I, Kawakami N, Miyashita K. Workplace Bullying and Psychological Distress: A Longitudinal Multilevel Analysis Among Japanese Employee. *J Occup Environ Med*. 2018;60(12):1067-1072.

3. Watai I, Nishikido N, Murashima S. Gender difference in work-family conflict among Japanese information technology engineers with preschool children. *J Occup Health*. 2008;50(4):317–327.

4. Watai I, Nishikido N, Murashima S. Development of a Japanese Version of the Work-Family Conflict Scale (WFCS), and examination of its validity and reliability. Sangyo Eiseigaku Zasshi. 2006;48(3):71-81. *(in Japanese).*

5. Shiomura K. Development of a web-based unconscious bias measurement tool: report on the need for the system and its overview of the web-based explore. Implicit site, multicultural communication studies. 2023;18:5-22 (in Japanese).

6. Greenwald AG, Nosek BA, Banaji MR. Understanding and using the implicit association test: I. An improved scoring algorithm. *J Pers Soc Psychol*. 2003;85(2):197–216.

7. psychexp.com. <https://psychexp.com>. Accessed November 10, 2024.

8. The Ministry of Health, Labour and Welfare. Guide to understanding current stress levels using the Brief Occupational Stress Questionnaire. <https://www.mhlw.go.jp/bunya/roudoukijun/anzeneisei12/dl/stress-check_j.pdf>. Accessed December 2, 2024.

9. Ali Jadoo SA, Aljunid SM, Dastan I, Tawfeeq RS, Mustafa MA, Ganasegeran K, et al. Job satisfaction and turnover intention among Iraqi doctors - a descriptive cross-sectional multicentre study. *Hum Resour Health.* 2015; 13:21.

10. Degen C, Li J, Angerer P. Physicians’ intention to leave direct patient care: an integrative review. *Hum Resour Health.* 2015; 13:74.

11. Yamazaki Y, Uka T, Marui E. Professional fulfillment and parenting work-life balance in female physicians in Basic Sciences and medical research: a nationwide cross-sectional survey of all 80 medical schools in Japan. *Hum Resour Health.* 2017;15(1):65.

12. Hetrick AL, Haynes NJ, Clark MA, Sanders KN. The theoretical and empirical utility of dimension-based work-family conflict: A meta-analysis. *J Appl Psychol*. 2024;109(7):987-1003.
